# Inuit parent perspectives on sexual health communication with adolescent children in Nunavut: “It's kinda hard for me to try to find the words”

**DOI:** 10.3402/ijch.v73.25070

**Published:** 2014-10-21

**Authors:** Gwen Healey

**Affiliations:** Qaujigiartiit Health Research Centre, Iqaluit, Nunavut, Canada

**Keywords:** Inuit, sexual health, public health, adolescents

## Abstract

**Background:**

For Inuit, the family unit has always played a central role in life and in survival. Social changes in Inuit communities have resulted in significant transformations to economic, political and cultural aspects of Inuit society. Where the family unit was once the setting for dialogue on family relations and sexuality, this has largely been replaced by teachings from the medical community and/or the school system.

**Objective:**

The purpose of this study was to explore Inuit parent perspectives on sharing knowledge with teenage children about sexual health and relationships.

**Method:**

A qualitative Indigenous knowledge approach was used for this study with a focus on Inuit ways of knowing as described in the *Piliriqattigiinniq* Community Health Research Partnership Model. Interviews were conducted with 20 individual parents in 3 Nunavut communities in 2011. Parents were asked about whether and how they talk to their children about sexual health and relationships. An analytical approach building on the concept of *Iqqaumaqatigiiniq* (“all knowing coming into one”), which is similar to “immersion and crystallization,” was used to identify story elements, groupings or themes in the data. The stories shared by parents are honoured, keeping their words intact as often as possible in the presentation of results.

**Results:**

Parents shared stories of themselves, family members and observations of the community. Fifteen of 17 mothers in the study reported having experienced sexual abuse as children or adolescents. Parents identified the challenges that they have and continue to experience as a result of forced settlement, family displacement and the transition of Inuit society. They expressed a desire to teach their children about sexual health and relationships and identified the need for emotional support to do this in the wake of the trauma they have experienced. Parents highly valued elders and the knowledge they have about family relationships and childrearing.

**Conclusion:**

There are powerful, unresolved healing issues in Inuit communities. The traumatic experiences of the settlement and residential school era continue to have an impact on present-day family relationships. To support parent–child dialogue on sexual health and relationships, parents identified a need to repair relationships between youth and elders, and to provide culturally sensitive support to parents to heal from trauma.

In 2009, Nunavut reported high rates of chlamydia and gonorrhoea, both of which are sexually transmitted infections, (3,772/100,000 and 1,588/100,000, respectively), compared to Canadians (259/100,000 and 33/100,000, respectively) (1). Concerns about these high rates and the high rates of teen pregnancy in Nunavut (161.3/1,000 compared to 38.2/1,000 in the rest of Canada) prompted community members in Nunavut to ask questions about how parents and their children talk about sexual health (2–4). The family unit was once the setting for dialogue on family relations, reproductive health and sexuality, and this has largely been replaced by teachings from the medical community and/or the school system. The purpose of this study was to explore Inuit parent perspectives on sharing knowledge with adolescent children about sexual health and partner relationships.

Family is the primary context in which a child grows, develops an identity, is socialized, is hurt and healed, and navigates physical and social development (5). The family is a naturally occurring unit and the context in which most behaviour-shaping experiences can occur. In recent years, increased attention has been given to the role of the family in predicting and understanding the sexual behaviour of adolescents in the literature (6–9). Family factors, such as communication, availability of parents, spending time together outside the home and engaging in activities together can have an impact on the extent to which behaviour problems or choices endure and become part of a healthy or unhealthy lifestyle (5, 7, 10). For example, adolescents who reported positive relationships and shared activities with parents were less likely to initiate sex (7). Parental communication about sex and condom use has been shown to directly relate to adolescent sexual behaviour (8). Whitaker and Miller (8) found that peer norms were more strongly related to sexual decision making among adolescents who had not discussed sex or condoms with a parent. The authors suggest that results indicate that a lack of communication may cause adolescents to turn to peers and that peers may then influence their behaviour. Parental discussions have been associated with less risky sexual behaviour among adolescents, less conformity to peer norms and a greater belief that parents provide the most useful information about sex (6, 8, 11). Research has shown that adolescents are more likely to use birth control when there is parental support to do so (12). In addition, research has shown that some teens want to have discussions about sex with their parents and other caregivers, more so than others, to help them understand sexuality and to guide them in their own decision making (13). Parent–teen discussions about sexual health topics are important because they (a) provide information to teens, (b) they reinforce parental values and (c) they buffer teens from peer pressure (8). Parental closeness and monitoring, rather than the actual specifics of parent–child communication, may also play a role because parents who talk to their children about sex or condoms may have already established closer relationships with their children (8, 11).

For Inuit, the family unit has always played a central role in life and in survival (14). Inuit kinship extends beyond familial affiliation to other non-biological affiliations including adoption, friendship, marriage or partnership, and namesake (15–18). Every person had a specific and essential role to play in making contributions towards family survival and the education of young children and adolescents (16, 19, 20). Before contact, small groups of Inuit families travelled together to different camps and hunting grounds, in *ilagiit nunagivaktangat*.^1^ Each person within a kinship group was valued for his or her contribution to the group's well-being and success. A child's earliest learning occurred as they observed and made meaning from the actions of their parents and extended family in the camp (22, 23). Children learned valuable behaviours, such as self-restraint, patience, non-aggressiveness, generosity and responsibility, by watching their family members lead by example (16, 24, 25).

When Inuit lived in family-based nomadic camps, teaching about sexual health and relationships was part of a dialogue between children and their parents or extended family, which occurred as part of the sharing of knowledge on a variety of topics. Painngut Peterloosie (26) highlighted the importance that was placed on the openness of the relationship dialogue between romantic partners in discussing, for example, menstruation, sex or sexual satisfaction. After the settlement era in the 1950s, during which time Inuit settled into communities, were sent to residential school and/or were sent away to Canadian cities for medical treatment, parent–child–extended family interaction changed significantly because many families were separated and displaced (21, 27, 28). Today in Nunavut, as in many other jurisdictions, parents and family are no longer the sole source for information about sexual health knowledge and behaviours, if they are a source at all (24, 29–33). The school system, peers, television, Internet, media, community members, teachers and others now play a role in the transmission of attitudes, knowledge and beliefs about sexual health behaviours (29, 33, 34). In a study of the perspectives of 53 Inuit women on teen pregnancy, some respondents identified less parental control over young people and greater influence on behaviour from other individuals outside of the family as a worrisome trend in larger communities compared to pre-settlement times (29). In a review of determinants of sexual health among Inuit adolescents, Steenbeek, Tyndall (32) asserted that Inuit parents and grandparents did not feel competent to instruct their own children in sexual health. Trauma experienced during and after the settlement and settlement era in the Eastern Arctic (35, 36); the loss of accumulated Inuit wisdom, knowledge, teachings and practices regarding life cycle, reproductive health and family planning that occurred as a result (21, 30, 32, 37, 38); and the changing nature of northern communities (28, 29, 39) could be factors contributing to the lack of confidence reported among parents.

## Methods

This qualitative participatory research study explored the topic of Inuit family communication about sexual health and relationships at the request of community members who participated in consultations conducted in Nunavut between 2006 and 2008 (2, 40). Their request was prompted by the high rates of sexually transmitted infections and high rate of teenage pregnancy in Nunavut communities compared to the Canadian population. The research project was designed and implemented in partnership with community wellness or research centres in each of 3 Nunavut communities. The researcher is from Nunavut and familiar with community and territorial research protocols. This study followed a modified grounded theory approach (41), which retains most of the defined characteristics of “classic” grounded theory, but takes a more subjective and reflexive stance which is more aligned with Indigenous knowledge and ways of knowing (42–44). The research framework focused on Inuit ways of knowing, specifically following the *Piliriqatigiinniq* Partnership Community Health Research Model (45). The model highlights 5 Inuit concepts, which informed the research approach: *Piliriqatigiinniq* (the concept of working together for the common good); *Pittiarniq* (the concept of being good or kind); *Inuuqatigiinniq* (the concept of being respectful of others); *Unikkaaqatigiinniq* (the philosophy of story-telling and/or the power and meaning of story); and *Iqqaumaqatigiinniq* (the concept that ideas or thoughts may come into “one”). A paper outlining the theoretical and methodological aspects of this study in greater detail is published elsewhere (45). Participants were engaged in the study through community health and wellness centres and were offered the opportunity to be project partners if they so desired. Inuit parents who had at least 1 teenage son or daughter between the age of 13 and 19 years were invited to participate. Interviews were conducted in a comfortable setting chosen by the participant, recorded with permission and transcribed verbatim. All questions were asked in English, and participants primarily responded in English. In the cases where they responded in Inuktitut, the author provided the translation and verified the translation with a third party. Participants were asked open-ended questions about their experiences talking about sexual health and relationships with their children and invited to tell stories and share experiences. Data were analysed through a process of “immersion and crystallization” (46) which, from the perspective of the researcher, is a process that is analogous to the Inuit concept of *Iqqaumaqatigiiniq*, “all knowing coming into one.” Through a process of listening to interviews, reading and re-reading transcripts and stories, themes crystalized in the data. A rigorous, respectful and mindful process was followed for the data analysis, which included: the comparison of findings to the known literature on the topic (47); reflexivity and bracketing of researcher perspectives before and during the study (48, 49); an iterative data collection and analysis process (50); discussion of findings with the local Nunavut-based advisors which included representatives from 2 community wellness centres,^2^ the Chief Medical Officer of Health for Nunavut, a Community Health Representative (CHR) and a public health nurse (50); reviewing the findings with participants or collaborators when and where appropriate (51); and honouring the stories, shared by parents, by keeping their words intact as often as possible in the presentation of results without breaching confidentiality (42, 45).

## Results

Twenty interviews were conducted in 3 Nunavut communities. The population of the communities ranged from 1,200 to 7,000. The respondents were aged between 30 and 58. Of the Inuit parents who volunteered to be interviewed for this study, 3 were fathers and 17 were mothers; 19 of 20 did not complete high school; 11 were employed in part-time, seasonal or casual work, 3 were unemployed and 6 were employed full-time. When asked about whether they spoke to their children about “sexual health,” parents described sexual health at the individual level as well as in the larger community and historic context. In response to the question about where they learned about sexual health, most mothers in the study disclosed being sexually abused as a child or adolescent. They stated that their experiences of child sexual abuse made them feel inadequate to talk to their children about sexual health. Both mothers and fathers shared a desire to teach their children about sexual health and relationships, and identified a need for support to help them do this, possibly by including elders. There were 2 primary themes in the data: (a) Parent–adolescent communication: “It's kinda hard for me to find the words” and (b) Bringing elders and young people together to talk about sexual health. Themes and quotes are presented in English, as that is the language in which the stories were conveyed, mirroring the way in which parents shared experiences.

### Parent–adolescent communication: “It's kinda hard for me to find the words”

Parents most often spoke of parent–adolesent communication in terms of what they perceived to be a struggle “between worlds” and how this struggle impacted relationships with their children. Parents in this study were among the first generation of Inuit born into permanent settlements. Their parents were often born and raised on the land in nomadic Inuit camps. The children of that era are the parents of today's youth generation. Participants spoke of the struggles families experienced adjusting to this “different world,” meaning the world of permanent settlements and the expectations of non-Inuit institutions, such as schools, nursing stations or the police force, in these new communities. When asked to explain the perceived divide between the parent and adolescent generations and impact on communication about sexual health, one father said,

When the kids are not listening to parents today maybe [it's] because the mother or the father is yelling to them. The child [becomes] too hard and it seems like they don't want to listen to the parents anymore. Because they yell … yeah, they yell too much. That they become hard. Hard and they will forget in their mind their childhood when they're older. So, some parents yell too much to the kids. Some parents are quiet. Some parents are keeping it [inside]. Different world now, different families. We all have different problems. Some people are [in a] very happy family. Some people are in not very good families. Some people are [in] very scary families. Some people are really not good – not welcoming people [in their] families. Like we're all different. – Father

Many of the parents in this study reported experiencing trauma, poverty and/or hardship in their childhood during this period of transition. Violence, substance use and/or mental illness are part of the pattern of ill-health in today's communities resulting from a lack of support to cope with those experiences. Parents described violence, substance use and unresolved trauma as factors that have perpetuated fractures in family relationships and in parent–adolescent communication about sexual health.

Parents in this study expressed a very strong desire to talk to their children about sexual health and relationships but questioned their confidence to teach their children. Fifteen of 17 mothers in the study disclosed experiences of sexual abuse in childhood or adolescence, and often described sexual health in terms of protecting their children from sexual abuse. Parents shared the stories to provide a context for explaining their desire to talk to their children about their negative or traumatic childhood and adolescent experiences in order to prevent their children from being similarly harmed. However, parents feared that they would be judged by their children for having engaged in the same behaviours that they are trying to prevent.

I've been on and off with a relationship with [my children's] father. And when we have our ups and downs – when he comes and goes like takes off and then – my daughter knows that – she knows I'm down and then I start telling her – I said when you're a teenager, don't ever get a boyfriend. I said don't ever get a boyfriend from here. Like you've got to find the right one and that's not abusive and like won't cheat on you and won't play games. So it's kinda hard for me to tell her more like, but I don't know how to explain it to her. So, I always try before I say anything I sit down and I think about – think about how – how – how am I going to say it to her. So, it's kinda hard for me to try to find the words. Yeah. And a way to say it to her.

Um, the way I see it – these young kids, now they're all shacked up and … at a young age. Like some of them are what? Thirteen – fourteen? And I'll say to myself, I could see myself when I was that young and like it's scary to get shacked up at a really young age and it's …. Because they're having kids. Are they just shacking up because they want to or … because I wonder – do they know about sexuality and life [relationships]? Do they know like once you're with the one – once you're with one girl or one boy you are just supposed to be together. Not to just do a couple of one night stands and then take off and then go to another girl …. That's the part that really scares me cause it's like they're getting that STI all the time and I know how it feels cause you have to take pills for that and then once you get treated and the next thing it happens – it goes back again. Same. Just like that circle of violence. It's like that. The same rotation over and over again. And they say they won't hurt you again. But the next thing it happens again.

Parents identified a need for greater emotional support to discuss sexual health and relationships with their children. Parents indicated that they struggled with how to talk to their children about sexual health because of traumatic experiences in their own youth. They identified a need for support for themselves and for each other in order to foster wellness in their own lives and in the lives of their children.

Definitely parents could be more involved [in talking to their children about sexual health and relationships] because it will not only help [us], but kids to be more aware of their surroundings. And what sexual preference they have and for them to respect themselves. And others, I think it would make a big difference if parents start talking to – they could do more talking to their children and not be shy about it. Because every parent has a role and to have brighter, healthier future they should talk to their kids.

### Bringing elders and young people together to discuss sexual health

In the context of parent–adolescent communication about sexual health, some parents talked about personal relationships among their parents’ generation – those who are now elders in the community. They spoke fondly of the older generation and provided stories and examples about the practices in which their parents had participated that are no longer followed today, such as arranged marriage. One participant indicated that the shift from the arranged unions of her parents’ generation to the self-selected partners of her teen daughter's generation was new for the family and something for which she was preparing.

It's changed a lot from [my parents] generation. Two parents – if there was a teenager, and the teenager was a boy and a girl … they would be set up – their relationship would be arranged. Once they reached puberty or once they get older, they would be living together. Then, even at the last minute– when they're ready to be together, there would be a marriage set up right away, early in the morning around 7 am right out on the land. And they would get married. Just like that. Not living with parents anymore, you just have to be with him. That's how some of them were. Our parents [generation]. That's how they used to be. So, I just really prepare for it – like as our ancestors used to do – prepare and all that. Looks like our teenagers are deciding who they want to marry. Who they want to be with. I just know my parents got married one day when they were 20 years old. – mother

These stories were shared to illustrate the rapid change in the formation of partner relationships within 3 generations in their communities. Participants talked about value they placed on the knowledge of elders about relationships and/or sexual health, and expressed a desire to see it revived and promoted among young people in the community. Parents indicated that while some adolescents may prefer to speak to elders or grandparents instead of their own parents, other young people may not yet be willing to listen to elders at all. In the latter situation, parents identified that the relationship between youth and elders needed to be restored. The parents felt that elders and youth were important supports for each other, and sometimes can communicate in a way that parents and youth cannot:

[Elders/grandparents] are not even trying [to talk to kids] anymore because … they won't listen. They're already listening to the music and the television and the Internet. And they don't want to listen to their elders. They know this. That's why [the older generation] shut their mouths. So, I guess what we need to develop is elders and young people together. Within the building, out there *gestures out the window*. And in the schools. Everywhere. On the land. When their friends are bothering them … or this young man or young lady wants to go out with one of my children … they don't tell me; they don't tell my wife. They always tell my mother (an elder). They talk to her. They are more open to them, than us as a parent.

## Discussion

The stories shared in this study illustrated, first, how parents related their trauma history to their understanding of parent–adolescent communication in today's communities in Nunavut. Parents described their childhood living in a “different world,” one in which families were separated and relationships were disrupted. They felt they did not have the confidence or “the words” to communicate with their children about sexual health and relationships as a result. Their stories highlight the loss of Inuit knowledge, teachings and practices regarding sexual and reproductive health that occurred as a result of the separation of families at that time (21, 30, 32, 37, 38).

Second, discussions about sexual health and relationships in the families of the participants, if they did occur, focused on teaching children to protect themselves from sexual abuse or abusive relationships. Data from the 2007–2008 Inuit Health Survey indicated that 41% of adult respondents in Nunavut (52% of women respondents and 22% of men respondents) experienced severe sexual abuse in childhood (52). Physical, emotional and psychological consequences of child sexual abuse can persist throughout the life course (53). Feelings of powerlessness and betrayal, anxiety, fear, post-traumatic stress disorder (PTSD) and suicidal ideas and behaviour have also been associated with a history of childhood sexual abuse (53–56). Shame, guilt, vulnerability, internal fragmentation, invalidation and cultural shame were some of the feelings reported by Indigenous women victims of sexual abuse in the literature that were also shared by participants in this study as having an impact on their ability to engage their children in a dialogue about sexual health and relationships (57). Previous research has shown that talking about child sexual abuse can be part of a therapeutic healing process for women, which is supported by the perspectives of the women in this study (58).

Third, parents highlighted the value that elders and their knowledge hold for them and in their community. They identified a desire to repair and support youth–elder relationships to foster dialogue on family, sexual health and intimate or personal relationships when parents are not able to be a support or resource. The parents’ vision of the role of elders in sexual health teaching reflects the Inuit kinship and family structure that was prominent before settlement. From their perspective, repairing that structure is an important part of promoting sexual health among adolescents. Previous research has shown that revitalizing Indigenous family and kinship perspectives, where they have been disrupted, is an important part of supporting positive, holistic parenting (59–62).

There are powerful, unresolved traumas and healing issues in Inuit communities related to the challenges Inuit have and continue to experience as a result of colonialism and the transition of Inuit society from one way of life to another (36, 63–67). The traumatic experiences of the settlement and residential school era continue to have an impact on present-day family relationships and parent–adolescent communication both in general and specifically about sexual health. Parents in this study identified a desire to move away from cycles of trauma and to be supported in engaging their children in dialogue about sexual health and relationships with a focus on revitalizing parent–adolescent and elder–youth relationships.

## Considerations and limitations

Only the perspectives of those with an interest in sharing their stories were represented in this study. The findings in this study are not representative of the entire population on the topic of sexual health, only the subset that had a story they wanted to share in 3 of 25 Nunavut communities. Given the historical and geographical differences between communities, there are a number of stories and perspectives on sexual health and relationships in Nunavut that could be explored in future research. In particular, future research should expand on this study to explore the perspectives of Inuit adolescents on the sources of knowledge about sexual health that they value as well as how to support survivors of child sexual abuse to have meaningful conversations with their children about sexual health.

## Conclusion

The results of this study highlight the importance Inuit parents place on engaging with children in a dialogue about sexual-health and relationships. Parents described events in the greater community and temporal context of Nunavut that they perceived to be barriers to communicating with their children about sexual health. They identified elders in their communities as supports for young people. This would be a positive contribution to the revitalization of Inuit kinship structure that existed before the displacement of families during settlement. The findings provide direction to public health programmes, services and practitioners to expand current strategies by including greater support for parent–child and elder–youth dialogue about sexual health and relationships in Nunavut. Healing and counselling services must be made available to families as part of this process, given the significant role child sexual abuse played in the lives of the parents in this study.

## Footnotes

1 *Inuktitut* terminology meaning, “a place used regularly or seasonally by Inuit for hunting, harvesting and/or gathering” (21).

2 The Arviat Community Wellness Centre and the 2nd declined to be named.
